# Potential miRNA Use as a Biomarker: From Breast Cancer Diagnosis to Metastasis

**DOI:** 10.3390/cells12040525

**Published:** 2023-02-06

**Authors:** Euclides Jordan-Alejandre, Alma D. Campos-Parra, Dora Luz Castro-López, Macrina Beatriz Silva-Cázares

**Affiliations:** 1 Posgrado en Ciencias Genómicas, Universidad Autónoma de la Ciudad de México, Ciudad de México 03100, Mexico; 2 Subdirección de Investigación Básica, Instituto Nacional de Cancerología, Ciudad de México 14080, Mexico; 3 Facultad de Ingeniería, Universidad Autónoma de San Luis Potosí, San Luis Potosí 78290, Mexico; 4 Coordinación Académica Región Altiplano, Universidad Autónoma de San Luis Potosí, San Luis Potosí 78760, Mexico

**Keywords:** breast cancer subtypes, microRNA, metastasis, diagnostic

## Abstract

Breast cancer is the most common cancer in women. Despite advances in diagnosis and prognosis, distal metastases occur in these patients in up to 15% of cases within 3 years of diagnosis. The main organs in which BC metastasises are the bones, lungs, liver, and brain. Unfortunately, 90% of metastatic patients will die, making this an incurable disease. Researchers are therefore seeking biomarkers for diagnosis and metastasis in different organs. Optimally, such biomarkers should be easy to detect using, preferably, non-invasive methods, such as using miRNA molecules, which are small molecules of about 22 nt that have as their main function the post-transcriptional regulation of genes. Furthermore, due to their uncomplicated detection and reproducibility in the laboratory, they are a tool of complementary interest for diagnosis, prognosis, and treatment. With this in mind, in this review, we focus on describing the most current studies that propose using miRNA independently as a potential biomarker for the diagnosis and prediction of brain, lung, liver, and bone metastases, as well as to open a window of opportunity to deepen this area of study to eventually use miRNAs molecules in clinical practice for the benefit of BC patients.

## 1. Introduction

Cancer is a pathophysiological disease characterised by cell-cycle dysregulation, and it was defined such as by the ancient Egyptians as early as 3000 B.C., who classified breast cancer (BC) as a “curse” [1]. Currently, the World Health Organization (WHO) defines cancer as a disease group originating from any organ or tissue where cells grow disproportionately, exceeding their limits and leading to an adjacent region’s invasion, spreading to other parts of the body. Further, under the genetic definition, multiple mutations in the genome are a common characteristic of this disease. These changes include the acquired genetic and epigenetic alterations that favour loss and gain of function, dedifferentiation, and the appearance of distinctive characteristics known as “cancer hallmarks” [2,3,4,5,6]. Being an ongoing health problem, the global cancer statistics show an estimated 19.3 million (1.2 million more than the 2018 estimation) new cancer cases and 10.0 million (400 thousand more than the 2018 estimation) cancer deaths. A total of 2.6 million BC cases were detected, and it is now the most diagnosed cancer worldwide. As the fourth-leading cancer cause of death, it accounts for 685,000 of all recorded cancer deaths, and it is exceeded only by lung cancer (with 1,800,000 deaths), liver cancer (830,000), and stomach cancer (770,000) [7,8]. The term BC indicates a malignant tumour that has developed in breast cells. It typically begins in the cells of the lobules, which are the milk-producing glands, or ducts, which are the ducts that drain milk from the lobules to the nipple. Approximately 5–10% of cancers are inherited from the mother or father and include mutations in genes involved in DNA damage repair, for example, BRCA1, BRCA2, PTEN, CHEK2, ATM, and PALB2. In contrast, 85–90% of BCs are considered sporadic cases, having a relationship with the patient’s lifestyle and their conditions, such as being obese, in late menopause, and being an alcoholic, among others [9,10]. The molecular classification of BC was based on the PAM50 signature, with clustering in the following groups: Luminal A (ER+ and/or PR+, HER2−, and Ki-67+ <20%), Luminal B (ER+ and/or PR+, HER2−, and Ki-67+ ≥20%), Luminal B-HER2+ (ER+ and/or PR+ and HER2+), HER2+ (ER-, PR-, and HER2+), HER2-enriched (ER+, PR+, and HER2+), and triple-negative (ER-, PR-, and HER2-). This classification is appropriate and valuable for a clinical oncologist to define the treatment that a patient will receive [11,12,13]. The treatment of tumours expressing estrogen (Er) and progesterone (PR) receptors consists of receptor inhibitors, such as tamoxifen, or aromatase inhibitors, such as letrozole, both of which avoid androgen-to-estrogen change. In HER2-enriched subtypes, trastuzumab manages a monoclonal antibody against the HER2 receptor. The therapy for triple-negative BC (TNBC) is chemotherapy, which involves alkylating agents such as docetaxel and paclitaxel. Patients with mutations in the BRCA1 and BRCA2 genes are treated with PARPS inhibitors such as olaparib and talazoparib [14,15,16,17]. Unfortunately, patients can develop resistance to treatment, which can lead to or be a consequence of metastatic development. Distal metastasis occurs in 10–15% of patients within the first 3 years, and only 5% of patients present distal metastasis at the time of diagnosis. Tragically, approximately 90% of patients with metastases will die [18]. The early identification of those patients who will develop metastases would be opportune. Many investigations have highlighted the RNA molecules, or the machinery related to their importance, involved in cancers [19,20,21,22,23,24,25,26,27,28,29]. Interestingly, with the appearance of microRNA (miRNA) and their involvement in different cancer-promoting processes, it has acquired interest for use as a potential biomarker for diagnosis, prognosis, recurrence, metastasis, and survival, and even as potential therapeutic targets, because of its ease of detection, as well as the ease of reproducibility in their analysis [21]. With this in mind, researchers have focused on the study of these molecules’ roles and their potential to promote cancer and metastasis. miRNAs are small non-coding RNA molecules with a length of 21 to 24 nucleotides, and their main function is the post-transcriptional regulation of genes, which includes long non-coding RNA (lncRNA), pseudogenes, and messenger RNA (mRNA) [30,31]. With this in mind, our proposal is to present the current information on the impact of miRNAs as a potential biomarker for BC diagnosis. For this purpose, in this bibliographical research, we focus on the miRNAs that predict metastasis in the bones, lungs, brain, and liver, independently, of BC patients.

## 2. miRNA Biogenesis

**Figure 1:**
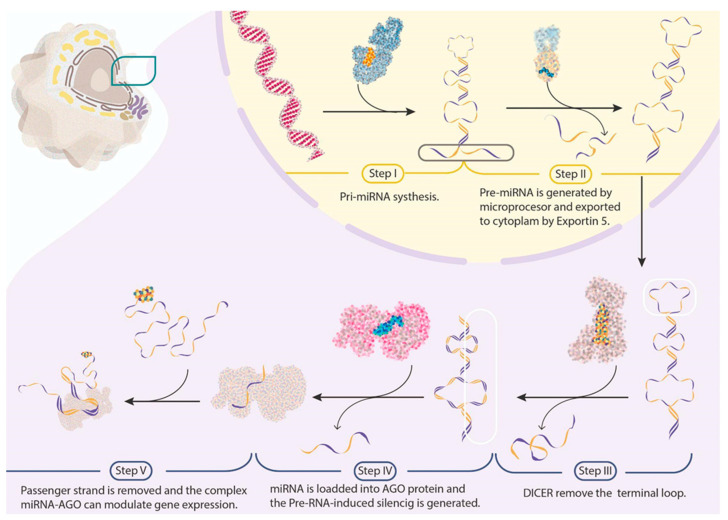
Canonical miRNA biogenesis. Schematic representation of the canonical pathway in miRNA generation, with the active sites proteins represented with different colours. We frame the RNA fragments that are processed in each part of the process.

miRNA molecules are small RNA molecules of 21 to 24 nt in size that regulate gene expression. Since their discovery, they have been potential biomarkers for cancer diagnosis, prognosis, treatment, and survival because of their greater stability than mRNA and their uncomplicated detection in tissues or plasma [32,33]. In addition, they may act as oncogenes or tumour-suppressive genes, depending on the cancer type and expression status [34,35,36,37,38,39,40,41,42,43,44,45,46,47,48,49]. The biogenesis of miRNA comprises canonical steps that involve: (I) Primary miRNA (pri-miRNA) synthesis by RNA Pol II or RNA Pol III—its structure comprises a 33–35 bp stem (divided into the lower and upper stem), a terminal loop, and 5′ and 3′ terminal single-stranded segments. (II) The microprocessor (Drosha and DGCR8) that cleaves the pri-miRNA at the lower stem by removing 11 bp and the 5′ and 3′ terminal segments—the molecule obtained is called pre-miRNA, and it is capable of being exported into the cytoplasm by exportin 5 and Ran-GTP. (III) In the cytoplasm, Dicer recognises the pre-miRNA structure and cuts the terminal loop. (IV) The RNA digested by Dicer should be loaded into an AGO protein, generating a pre-RNA-induced silencing complex (RISC). (V) The passenger-strand removal (the strand complementary to the miRNA sequence, or the guide strand) occurs upon the pre-RISC formation. Mature RISCs can modulate gene expression through binding to miRNA response elements, which are typically localised in the 3′ UTR mRNA sequence (we schematise the canonical steps in Figure 1) [50,51,52]. With this in mind, the existing knowledge about miRNA has resulted in the creation of miRBase, a digital library with 38,589 harping precursors and 48,860 mature miRNAs from 271 organisms. This library provides related information, such as the nomenclature of novel miRNAs, sequences, biogenesis precursors, loci, sequencing information, and cellular functions [53]. Another interesting database is plasmiR, which systematically provides experimental evidence of the diagnostic and prognostic potential of circulating miRNAs against human diseases [54]. Moreover, version 3.0 of the Human MicroRNA Disease Database (HMDD v3.0) manually collects a significant number of miRNA disease-associated entries from the literature, with increased precision based on the literature-derived evidence code, resulting in six generalised categories (genetic, epigenetic, target, circulation, tissue, and other) covering 20 types of detailed evidence codes. Since biomedical research continually demands comprehensive databases of microRNA–disease associations, this last database is a strong choice [55].

## 3. miRNAs as a Potential Biomarker for Breast Cancer Diagnosis

It is important to highlight that actual early BC diagnosis is uncomfortable, using techniques ranging from mammography and breast ultrasound, which are considered less invasive, to biopsies. These protocols present a high rate of false positives due to the density of fibroglandular breast tissue, which increases the probability of camouflaging underlying BC on a mammogram, given that both conditions appear white [56]. In this context, a complementary molecular tool that helps with achieving a good diagnosis and staging of the disease is a less-invasive technique. In this sense, researchers have taken advantage of genomics, bioinformatics, and molecular biology tools to find miRNAs that provide valuable diagnostic information [57]. For example, Li et al. identified five types of miRNAs (Let-7b-5p, miR-122-5p, miR-146b-5p, miR-210-3p, and miR-215-5p) in the plasma samples of BC patients (in contrast to normal controls) using the exiqon miRNA qPCR panel to screen by phase, and then they further validated their results with qRT-PCR. The ROC correlation result of miRNA expression in both samples was 0.978, supporting the use of this miRNA panel as a promising biomarker in the diagnosis of BC [58]. Likewise, Orangi and Motovali-Bashi reported the use of miR-9 and miR-34a as diagnostic biomarkers in BC. Their study demonstrated that the downregulation of both molecules could be related to tumour detection and even to stage in BC tumour samples [59]. Moreover, Bakr et al. described the use of miR-373 as a diagnostic biomarker in BC patients. In addition, they evaluated its target genes (VEGF and cyclin D1), demonstrating that miR-373 acts as an oncomir, and it would be a fundamental biomarker for BC diagnosis and prognosis by VEGF and cyclin D1 targeting [60]. On the other hand, Zhang et al. examined miR-26b-5p, miR-106b-5p, miR-142-3p, miR-142-5p, miR-185-5p, and miR-362-5p upregulation in BC patients. Through the area under the curve (AUC) results that were obtained with ROC analyses, they constructed two panels and observed a higher AUC (0.957) in miR-185-5p and miR-362-5p, with a sensitivity of 92.65% and a specificity of 92.31%. The investigation revealed that the miRNA panel was capable of differentiating BC patients from healthy controls, and it even impacted numerous cancer-related pathways [61]. Further, Lv et al. outlined a relationship between the downregulation of miR-145 and BC diagnosis, independent of cellular subtype. They performed a meta-analysis of eight articles whose quantitative methods used qRT-PCR. They showed that miR-145 expression was −2.57 in BC tissues versus normal breast tissues. The study reported a negative relationship between miR-145 expression and histological tumour grade, suggesting the potential of miR-145 to be used as a biomarker [34]. In addition, Kahraman et al. studied and highlighted the diagnostic role of seven miRNA molecules (miR-126-5p, miR-144-5p, miR-144-3p, miR-301a-3p, miR-126-3p, miR-101-3p, and miR-664b-5p) with high values of specificity (74.2%), sensitivity (83.8%), accuracy (79%), and AUC (0.814) in TNBC. The authors used RT-qPCR to study 63 blood samples from women (21 patients with TNBC diagnoses and 21 healthy patients), demonstrating the high expression of miR-126-5p as one primary tumour-suppressive microRNA in BC, and it was also the most significant miRNA for diagnosis in the analysed panel [62]. Further, Wang et al. performed a meta-analysis to study different biomarkers in BC. They examined 11 reports that used regular detection methods such as ELISA assay and qRT-PCR. The investigation identified a higher miR-1246 and miR-21 expression in BC plasma samples. Equally important, Hannafon et al. demonstrated the differential expression between 101-196 miRNA molecules. Their experimental results highlighted that miR-1246 and miR-21 present potential diagnosis roles in BC, and they observed the same results in ROC analyses (AUC, 0.69), with the highest combined value (0.73), highlighting their fair predictive power. Likewise, Li et al. studied BC diagnosis improvement through an miR-21 and miR-27a panel. They observed higher plasma expressions for both miRNAs, correlating them with histological grade, clinical stage, and lymph node metastasis. Further, they analysed the potential diagnostic role of both miRNA molecules, and their ROC curve analyses demonstrated results of 0.737 and 0.771, respectively; meanwhile, their sensitivity, accuracy, and negative predictive values were 94.57%, 88.27%, and 83.72%, respectively. With these results, the authors discussed a significant improvement in current BC diagnosis through plasma miR-21 and miR-27a [63,64,65]. Further, a four-phase validation study by Zou et al. found 12 miRNAs types (let-7b-5p, miR-106a-5p, miR-19a-3p, miR-19b-3p, miR-20a-5p, miR-223-3p, miR-25-3p, miR-425-5p, miR- 451a, miR-92a-3p, miR-93-5p, and miR-16-5p) in a panel of serum samples from 216 BC patients (versus 214 normal control subjects) using an exiqon miRNA qPCR panel, and they validated the results with qRT-PCR. This panel of miRNA molecules detected in serum has the potential to be used in the non-invasive diagnosis of BC [66]. Likewise, Chen et al. analysed the efficacy of a panel of three miRNA types (miR-9-5p, miR-34b-3p, and miR-146a-5p) for the diagnosis of invasive BC carcinoma in 260 participants. The study indicated superior results for ROC analysis (AUC of 0.880), with 86.25% sensitivity and 81.25% specificity. Further, they found a relationship between this miRNA panel and the Rap1, MAPK, Wnt, and cGMP-PKG signalling pathways. Their investigation concluded with the panel’s detection in serum. In addition, the authors highlighted its use (Table S1), such as serving as non-invasive biomarkers, for the diagnosis of invasive BC carcinoma [67]. There is a wide variety of miRNA molecules with the potential to be used as biomarkers (Table 1).

## 4. Breast Cancer Metastasis

**Figure 2:**
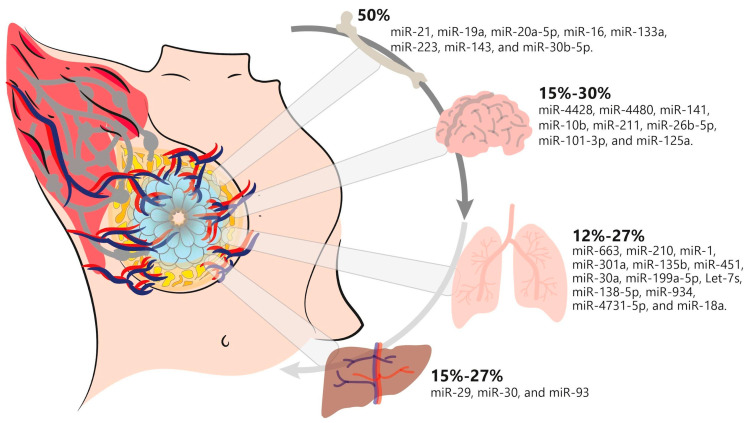
miRNAs associated with metastasis to different organs in breast cancer. The percentage of the incidence of miRNAs implicated in BC metastatic process to the principal organs affected is summarised, with bone being the most-affected organ and the liver and lungs occupying the lowest incidence rates.

It is important to consider that BC metastasis is a process described as spread via regional lymph nodes to distal and proximal tissues such as the bones, lungs, brain, and liver. Indeed, the rates and sites of distal metastasis can vary depending on age and stage of diagnosis. Further, metastasis is a sequential process that results from the evolutionary acquisition of characteristics that let tumour cells from a primary site dissociate, disseminate, migrate, survive, extravagate, and infiltrate a distant site to form a metastatic niche, triggering them to survive and proliferate in a new environment. To disseminate tumour cells from the primary site to the circulation, circulating tumour cells (CTCs) play a significant role. These cells undergo EMT to invade the extracellular matrix at the primary site. Eventually, to survive anoikis and immunosurveillance, primary tumour cells and CTCs discharge RNAs and miRNAs condensed in exosomes, which promote the survival of metastatic cancer cells at the metastatic site. In addition, for the invasion and colonisation of secondary tumour sites, cellular adaptability is crucial for these processes, accompanied by different signalling pathways, for example, the epithelial–mesenchymal transition (EMT), OPG/RANK/RANKL, TGF-β, IGF system, PI3K/Akt/mTOR, Wnt, Hippo, HIF-1, TNF, FoxO, JAK/STAT, PD-1/PD-L1, and EGFR pathways, among others. It is important to highlight that several miRNA molecules regulate these pathways, focusing their importance for the disease [84,85,86,87]. Since 2007, miRNAs have been associated with the sequential metastasis steps in BC. The first miRNA studied in mouse models, cell lines, and patients was miR-10b. In their investigation, the authors demonstrated its higher expression in metastatic BC [88]. Moreover, various investigations were published describing miRNAs associated with BC metastasis, and it was named as an activator of metastasis. miR-29a, miR-103/107, miR-155, miR-7, the miR-200 family, miR-205, miR-448, miR-181, and miR-661 have been associated with the EMT process, cell invasion, and proliferation in BC [89,90,91]. Interestingly, in recent years, researchers have made efforts to determine that BC exhibits metastatic heterogeneity with distinct metastatic precedence to various organs, resulting in distinctions in prognosis and responses to treatment in BC patients. Generally, bones, the lungs, the brain, and the liver are the focal places for BC metastases. However, the underlying molecular mechanism of this metastatic heterogeneity remains to be explained [92,93]. For this purpose, in this review, we pay special attention to distinguishing those miRNA types that could predict metastasis in the primary organs metastasised by BC, as described below. As shown in Figure 2, we summarise some important metastatic miRNA types in BC for different organs.

### 4.1. miRNAs Involved in Breast Cancer Bone Metastasis

Bone is the primary tissue involved in metastasis in BC patients, with a prevalence of up to 50%, and it continues to be the most common metastatic site in the luminal A and B and HER2 subtypes. Bone metastasis is an incurable complication that causes patients to suffer pain, spinal cord compression, fractures, and hypercalcemia [94]. In this process, miRNAs do not go unnoticed. As an overview, we describe several examples below. Recent evidence suggests that BC-derived miRNAs play a primary role in tumour metastasis via exosome transfer. Yuan et al. reported that the miR-21 levels in the serum exosomes of BC patients with bone metastasis were significantly higher than those in patients without bone metastasis. The researchers noticed in mouse xenograft models that exosomes containing miR-21 secreted by BC cells contributed to creating a pre-metastatic niche by promoting osteoclast differentiation and enhancing bone metastasis through PDCD4 regulation. These findings propose that BC-cell-derived exosomes play an important role in promoting BC bone metastasis, which is associated with pre-metastatic niche formation by transferring miR-21 to osteoclasts [95]. Bone matrix integrin-binding sialoprotein (IBSP) and exosomal miR-19a are overexpressed in ER+ BC patients with bone metastases. In addition, the study by Wu et al. showed that miR-19a enhances the IBSP function in bone, inducing osteoclastogenesis through a favourable microenvironment creation for bone colonisation. Thus, both molecules could be two effective biomarkers of ER+ BC to predict the risk of bone metastasis [68]. In another similar report, Guo et al. focused on the analysis of miRNA from BC tumour-cell-derived exosomes to investigate the communication between the bone microenvironment and tumour cells, where miR-20a-5p stood out. The results indicate a higher expression of miR-20a-5p in breast tumour tissues and in exosomes from MDA-MB-231 cells. They also showed that exosomes derived from MDA-MB-231 cells transferred miR-20a-5p to murine bone marrow macrophages (BMM) and facilitated osteoclastogenesis through SRCIN1 targeting. The authors proposed that their findings could be applied to develop a therapeutic intervention targeting exosomes or miR-20a-5p in BC progression [69]. In another interesting study, Kitayama et al. created an in vivo bone metastasis model for histological and morphometric evaluation in which they transplanted miR-16-, miR-133a-, and miR-223-transfected MDA-MB-231 cells into the proximal tibia of nude mice. The authors observed that the osteolytic expression factors, such as receptor activator of nuclear factor-κB receptor ligand (RANKL), interleukin (IL)-1β, IL-6, parathyroid hormone-related protein (PTHrP), and tumour necrosis factor (TNF), increased in the miR-16 group. Meanwhile, in the miR-133a and miR-223 nude transgenic mice, its expression decreased. Moreover, in vitro assays showed that miR-16 overexpression increased osteoclast activity and bone destruction in MDA-MB-231 cells, whereas in MDA-MB-231 cells transfected with both miRNAs, opposite results were obtained. Thus, the authors concluded that miR-16 promoted the osteoclast activities and bone destruction caused by BC metastasis in the bone microenvironment, whereas miR-133a and miR-223 suppressed them [70]. On the other hand, miR-143 is involved in bone metastasis, playing a tumour-suppressive role, and being down-regulated in BC patient tissues and in BC cell lines. In this regard, Du et al. demonstrated that its expression inhibits bone metastasis through the downregulation of Jag1 and, consequently, it inhibits GTPAsa Rho signalling, leading to an increase in apoptosis and decreasing the migration rate and invasion of BC cells to 75%. The authors discussed that miR-143 has an anti-metastatic effect on BC growth and thus may be beneficial in the treatment of this disease [71]. Further, Estevão-Pereira et al. analysed the expression of miRNAs in primary versus metastatic tumour tissues. The study revealed that miR-30b-5p was significantly expressed in metastatic tissues and, moreover, it was observed that the expression of this miRNA was higher in the liquid biopsies of patients with advanced versus early disease. Thus, the authors proposed miR-30b-5p, as a potential biomarker of disease progression, as a useful tool for patient follow-up [72]. All of these results suggest that miRNAs have the potential to operate as a biomarker of BC bone metastasis; however, further investigations are needed, especially in large patient cohorts.

### 4.2. miRNAs Involved in Breast Cancer LungMetastasis

The lung is another organ susceptible to metastases by BC. The lung metastasis incidence is between 12 and 27%, with basal BC responsible for the metastasis to this organ [96]. There are studies that have examined the molecular mechanisms that lead to this. For example, a valuable study by Zhang et al. demonstrated the significant importance of eight miRNA types (miR-663, miR-210, miR-1, miR-301a, miR-135b, miR-451, miR-30a, and miR-199a-5p) that can predict metastasis to the lungs in BC patients. This study was the first to establish and validate a lung metastasis prediction nomogram using the METABRIC and TCGA databases. These authors provided a reliable assessment tool for clinicians to help select a treatment. Thus, relying on this model, clinicians and professionals may assess the risk of lung metastasis in BC patients, selecting appropriate medical considerations and optimising the therapeutic regimen [97]. Another report evidenced the role of Lin28B as a precursor of a pre-metastatic niche that promotes metastasis to the lungs in BC patients. This allows for neutrophil recruitment, which is necessary for immune suppression in pre-metastasised lungs by up-regulating PD-L2, with a deregulated cytokine milieu. Interestingly, these authors also identified that exosomes released from BC stem cells with low let-7d levels are necessary for Lin28B-induced immune suppression. Therefore, high levels of Lin28B and low levels of let-7s have the potential to predict poor prognosis and lung metastasis [98]. On the other hand, exosomal miR-138-5p derived from BC cells inhibited M1 polarisation and promoted M2 polarisation by inhibiting KDM6B expression in macrophages. Exosomal miR-138-5p-treated macrophages promoted lung metastasis, and the circulating exosomal miR-138-5p level had a positive correlation with BC progression. Therefore, exosomal miR-138-5p represents a promising prognostic marker and target for BC treatment [73]. In addition, Lu et al. used a lung metastasis model to demonstrate the role of miR-934 in BC metastasis. They demonstrated, in lung metastatic tissue, an association between miR-934 upregulation and poor prognosis. In fact, the miR-934 downregulation inhibited the migration and invasion process in BC cells through a negative correlation with PTEN and EMT. In addition, the investigators observed BC metastasis to the lung promotion through miR-934 by regulating different pathways, such as N-glycan biosynthesis, the mTOR signalling pathway, and endocytosis [74]. Further, an investigation by Lang et al. revealed the role of the miR-4731-5p/PAICS/FAK axis in BC metastasis. They analysed the axis using nude mouse xenograft tumour tissues and in vitro assays. Their report demonstrated miR-4731-5p’s inhibitory role in BC cell glycolysis and EMT transition through PAICS-induced FAK phosphorylation reduction [75]. Indeed, Krutilina et al. observed the downregulation of miRNAs encoded by MIR17HG in MB231RN-LM cells, a cellular subline of MDA-MB-231, isolated from spontaneous lung cells and generated from tumour cells orthotopically implanted in a mammary fat pad. Of these miRNA molecules, miR-18a stood out, as upon its overexpression, they observed a tumour growth decrement and tumour metastasis suppression, in part through the direct regulation of hypoxia-inducible factor 1α (HIF1A) activity. These data described that miR-18a downregulation, as well as concomitant HIF1A activity increment, could be essential for promoting the metastasis of basal breast cancer to the lungs [76]. Remarkably, we observed limited studies focused on describing and studying BC miRNAs as a predictor of lung metastasis. We posit that there is a need for further deep investigations on this topic.

### 4.3. miRNAs Involved in Breast Cancer Brain Metastasis

With an incidence rate of between 15 and 30%, the brain is the third-most-compromised organ by BC metastases. Brain metastasis is associated with poor prognosis and affects quality of life. It occurs in triple-negative and HER2+ BC patients, with a median survival of only 4–14 months. In contrast, luminal cancer types typically have lower brain metastasis rates [99]. This phase is complex and involves metastatic niche formation. In this context, miRNA plays an important role in that process; despite knowing this, we do not yet understand this process, and efforts to find a potential miRNA as a biomarker continue. For instance, Sato et al. evaluated serum miRNA profiles in patients with BC, with and without brain metastasis, using high-sensitivity microarrays. Their results exposed two miRNA molecules, miR-4428 and miR-4480, which could significantly distinguish patients with brain metastasis, with 0.779 and 0.781 AUC values, respectively, demonstrating that miRNA may be useful as a biomarker for predicting brain metastasis in patients with BC [77]. Ahmad et al. explored miR-10b as a potential biomarker of metastasis to the brain in BC patients. They observed a higher and significant expression in tumour tissues from BC patients who had metastasised to the brain vs healthy samples. Likewise, by in vitro analysis, they demonstrated that miR-10b stimulates the invasive capacity of BC cells. The authors highlighted miR-10b as a therapeutic target against brain metastases [78]. In another study, Pan et al. established in vitro and in vivo experimental models of TNBC brain metastasis to investigate and characterise the functional role of miR-211. Their results validated that miR-211 downregulated SOX11 and NGN2, which drive early and specific brain colonisation through enhancing migration across the blood–brain barrier (BBB), as well as BBB adhesion and tumour cell stem properties to promote metastasis to the brain. These analyses identified a high level of miR-211 in plasma, predicting the brain metastasis of TNBC in in vivo mouse models [100]. Further, Harati et al. studied the role and molecular regulation of COX-2 in BC cell extravasation in brain metastasis. They found that miR-26b-5p and miR-101-3p downregulation led to a synergistic effect of COX-2/MMP-1 expression, enhancing BC transendothelial migration to the brain endothelium. The authors demonstrated that the overexpression of both miRNA types targeted COX-2, with this interaction enhancing trans-endothelial migration suppression. Thus, the study observed the use of both miRNA types as therapeutic molecules [79]. In addition, Fu et al. analysed circBCBM1 overexpression (a circular RNA) in the brain metastatic cells, tissue samples, and plasma from BC patients. Such overexpression was associated with shorter brain-metastasis-free survival (BMFS) in BC patients. Mechanistically, circBCBM1 acted as an miR-125a sponge to inhibit its activity, resulting in the overexpression of bromodomain-containing 4 (BRD4) and the subsequent upregulation of matrix metallopeptidase 9 (MMP9) via the Sonic Hedgehog signalling pathway. The authors deduced that circBCBM1 may operate as a potential therapeutic target for brain metastasis from BC [80]. In addition, Yoo et al. investigated and reported on the application of MN-anti-miR10b, an RNAi-targeted therapy type, to a metastatic BC model in the brain. The authors demonstrated the therapy delivery into metastatic lesions and obtained evidence of a therapeutic effect manifested as the inhibition of metastatic progression. This research represents a further step toward the translation of similar RNAi-directed therapies for the systemic treatment of metastatic disease. Thus, a potential candidate system for the treatment of BC metastasis was presented [81]. Debeb et al. used brain metastasis mouse models to evaluate the role of miR-141, and they observed that the overexpression of this miRNA-enhanced brain metastatic colonization and high miRNA serum levels associated with shorter BMFS. Thus, the authors discussed that miR-141 should be tested as a biomarker or potential target to avoid brain metastasis [82]. In sum, all these findings suggest that miRNAs have the potential to operate as a therapeutic candidate for biomarkers or as a therapeutic target for BC brain metastasis.

### 4.4. miRNAs Involved in Breast Cancer Liver Metastasis

According to statistical data, the liver metastatic incidence rate is 15–27%, thus being the fourth-most-compromised organ by BC metastases. To understand miRNA behaviour in this context, Shibuya et al. established a mouse model of BC-patient-derived tumour xenografts in which cancer stem cell (CSC) marker CD44+ cancer cells metastasised in the liver. Likewise, in that same mouse model, they analysed miR-25, miR-93, and miR-106b deregulation. They observed the relationship between miR-93 overexpression, invasiveness, and 3D-organoid-forming ability suppression in BC cells, triggering its liver metastatic potential suppression. Taken together, their findings submitted that miR-93 function, such as metastasis suppression, inhibited the invasiveness and properties of CSCs in BC [83]. An equally important study performed by Shastri et al. evaluated the impact of caloric restriction on BC metastasis progression, triggering the increment of miR-29 and miR-30 expression in both normal and metastasised liver tissues. The authors’ findings proposed a dietary alteration role in liver metastasis treatment; however, their conclusions require deeper evaluation [101]. In concordance with our bibliographical research on lung metastasis, we found limited reports on miRNA being used in the BC prediction of metastasis to the liver. We questioned whether this limit is related to difficulties in accessing this organ, highlighting the need for the investigation and development of new study approaches to understand the relationship between BC and its liver metastasis through miRNA.

## 5. Conclusions and Perspectives

Breast cancer is the most common cancer among women worldwide. This disease is curable in 70–80% of cases of early disease and non-metastatic disease. However, the difficulties or false positives during diagnosis and metastasis development to distant organs caused by this disease are the primary difficulties to be overcome. The main organs to which BC metastasises are bones, the lungs, the brain, and the liver, which are life-threatening to patients during BC progression [56]. Nevertheless, as reviewed, BC patient management continues to be a clinical challenge. In this regard, the scientific community continues to search for additional biomarkers that can be used in clinical practice to facilitate an oncologist’s management of a patient. For this purpose, we recapitulated and presented experimental evidence that supports the use of miRNA in cancer and BC. miRNAs have acquired much attention due to their ease of detection and reproducibility, and as we have summarised, they may play a potential role in future diagnosis and as a predictor of metastasis to the primary organs metastasised by BC. Despite this, we presented miRNA expression heterogeneity and its impact on cancer depending on its type; the most relevant element of this compilation was that we observed that the miRNA found to predict metastasis to bones, lungs, liver, and brain in BC patients were exclusive. That is, according to this compilation of information, miRNAs are organ-specific. Most interestingly, we found reports in which the researchers detected miRNA in liquid biopsies, using it as a predictive biomarker of metastasis and favouring a basic biomarker principle—its lower invasiveness. In alignment with this, the investigations demonstrated that principal miRNA detection was in exosomes, and consistent with this principle, the sampling was easier for the patient and was less invasive and aggressive. However, herein, we identified and presented in silico, in vitro, and in vivo studies and meta-analyses that confirm the use of miRNA in clinical trials for new therapeutic development strategies. Our investigation summarised some miRNA types proposed as diagnostic biomarkers and predictors of metastasis to different organs. Nevertheless, the question arises: which of these is worth evaluating in large cohorts, or in multi-centre studies, to recommend and use as a potential biomarker? It is important to highlight that different authors have framed important characteristics to consider for miRNAs to be used as molecules for clinical implementation, of which the following stood out: specificity, sensibility, and non-invasiveness. Further, their results, although encouraging, need to be studied to confirm their consistency in therapies focused on human treatment. A critical limitation in these studies is the population biases on which the researchers based their work, primarily because of the genetic background of the participants analysed. However, in many of these reports, we identified consistency in certain specific characteristics associated with the disease, such as size, stage, and BC cellular subtype. Therefore, this research opens a window of opportunity to further analyse the miRNA types that are predictive of metastasis in large cohorts.

## Figures and Tables

**Table 1:** Impact of microRNAs on cancer-derived metastatic phenotype.

miRNA	Study Type	Samples Number	Samples Type	Samples Sources	Detection Methodology	AUC	Sensitivity	Specificity	Potential as	Reference
Let-7b-5p, miR-122-5p, miR-146b-5p, miR-210-3p and miR-215-5p	Multi-phase validation	289 BC samples and 257 normal controls	Blood samples	Hospital of Nanjing Medical University.	exiqon miRNA qPCR	0.843	81.1%	NR	Diagnosis	Li et al. [58]
miR-9 and miR-34a	Case–control	31 tumour tissues, 31 adjacent non-tumour, and 20 healthy controls	Tissues	National Tumor Bank and Isfahan Cancer Research Center of Seyed-o-Shohada Hospital.	qRT-PCR	0.71 and 0.72	83.33% and 72%.	70.37% and 76%	Diagnosis	Orange and Motovali-Bashi [59]
miR-185-5p and miR-362-5p	Case–control	68 BC patients and 13 controls	Blood samples	Qilu Hospital of Shandong University.	qRT-PCR	0.957	92.65%	92.31%	Diagnosis	Zhang et al. [61]
miR-126-5p, miR-144-5p, miR-144-3p, miR-301a-3p, miR-126-3p, miR-101-3p, and miR-664b-5p	Clinical trial	21 patients with TNBC diagnosis and 21 healthy	Blood samples	Medical Faculty of the Friedrich-Alexander University Erlangen-Nürnberg.	qRT-PCR	0.814	83.8%	74.2%	Diagnosis	Kahraman et al. [62]
miR-1246 and miR-21	In vitro and Case–control	16 BC samples and 16 healthy samples	Exosomes from plasma	USA	Small RNA sequencing and qRT-PCR	0.73	NR	NR	Diagnosis	Hannafon et al. [63]
miR-1246 and miR-21	Meta-Analysis	11 reports	Peripheral blood samples	NR	ELISA and qRT-PCR	NR	NR	NR	Diagnosis and prognosis	Wang et al. [64]
miR-21 and miR-27a	Case–control	129 BC patients and 50 patients with benign breast lesions as control group	Fasting venous blood	Jinan People’s Hospital Afliated to Shandong First Medical University.	qPCR	0.737 and 0.771,	90.62%	74.00	Diagnosis	Li et al. [65]
Let-7b-5p, miR-106a-5p, miR-19a- 3p, miR-19b-3p, miR-20a-5p, miR-223-3p, miR-25-3p, miR-425-5p, miR- 451a, miR-92a-3p, miR-93-5p, and miR-16-5p	Four-phase validation study	216 BC patient samples and 214 normal control samples	Blood samples	Jiangsu Provincial People Hospital.	exiqon miRNA qPCR validated by qRT-PCR	0.941	0.872	0.893	Diagnosis	Zou et al. [66]
Bone metastasis										
miR-19a	In vitro and in vivo	NR	BC tissue from bone-metastatic lesions and primary BC tissue	NR	Taqman PCR	NR	NR	NR	Promoting metastasis	Wu et al. [68]
miR-20a-5p	In vitro	NR	MCF-7 and MDA-MB-231 cell lines	NR	qRT-PCR	NR	NR	NR	Promoting migration and invasion	Guo et al. [69]
miR-16, miR-133a, and miR-223	In vitro and in vivo	NR	MDA-MB-231 cell line, bone, and tibia samples	NR	qRT-PCR	NR	NR	NR	Promoting osteoclast activities and bone destruction	Kitayama et al. [70]
miR-143	In vitro	15 patients	BC tissue, normal adjacent tissues, MDA-MB-231, MDA-MB-436, SK-BR3, CAMA-1, and normal MB 157 cell lines	Fudan University Shanghai Cancer Center.	qRT-PCR	NR	NR	NR	Tumour suppressor	Du et al. [71]
miR-30b-5p	Cohorts	20 localized tumours and 25 advanced disease	Liquid biopsies	Breast Cancer Clinic and Laboratory Medicine Department of the Portuguese Oncology Institute of Porto.	qRT-PCR	0.831	88.9%	66.7%	Progression biomarker	Estevão-Pereira et al. [72]
Lung metastasis										
miR-138-5p	In vitro and in vivo	NR	Lung tissues	Chinese PLA General Hospital.	qRT-PCR	NR	NR	NR	Promotion of lung metastasis and progression	Xun et al. [73]
miR-934	Case–control and in vitro	50 pairs of frozen BC tissue, adjacent normal tissue, and 21 lymph node metastasis BC tissue samples	Lung metastatic tissue	Huai’an Maternity and Child Health Care Hospital.	qPCR	NR	NR	NR	Prognosis biomarker	Lu et al. [74]
miR-4731	Cn vitro and in vivo	50 patients	Tumour tissue, MDA-MB-436, MDA-MB-453, MCF-7, and MDA-MB-231 cell lines	Chongqing University Central Hospital.	qRT-PCR	NR	NR	NR	Inhibition of EMT transition	Lang et al. [75]
miR-18a	In vitro and in vivo	NR	Primary tumour and lung tissues	NR	qPCR	NR	NR	NR	Promotion of metastasis	Krutilina et al. [76]
Brain metastasis										
miR-4428 and miR-4480	Case–control	51 samples brain metastasis and 28 samples without brain metastasis	Serum	National Cancer Center Hospital. Japan.	Microarray	0.779 and 0.781 respectively	82.4% and 76.5%	f 64.3% and 71.4%	Prediction of brain metastasis	Sato et al. [77]
miR-10b	Retrospective Case–control	20 samples with brain metastasis and ten samples without brain metastasis	Tumour tissue block	Department of Pathology, Karmanos Cancer Institute, Wayne State University.	qRT-PCR	NR	NR	NR	Biomarker of brain metastasis and a potential therapeutic target	Ahmad et al. [78]
miR-26b-5p and miR-101-3p	Pre-Clinical	NR	Brain metastatic tumours and primary tumours	NR	qRT-PCR	NR	NR	NR	Prognostic and therapeutic	Harati et al. [79]
miR-125a	Cohort	Cohort (I) 13 pairs of BC and adjacent normal breast tissues Cohort (II) Six BC brain metastasis (BCBM) tissues Cohort (III) 20 BC and 20 BCBM patients’ plasma samples, and Cohort (IV) 53 BCBM patients’ primary tumour tissues.	Tissues and plasma	Liaocheng People’s Hospital.	qRT-PCR	NR	NR	NR	Therapeutic target	Fu et al. [80]
MN-anti-miR10b	In vitro and in vivo	NR	NR	NR	Bioluminescence and microscopy	NR	NR	NR	Treatment in brain metastasis	Yoo et al. [81]
miR-141	In vitro and in vivo	10–15 mice per group	Brain metastasis tissues	NR	RT-PCR and fluorescence methods	NR	NR	NR	Biomarker and therapeutic target of brain metastasis	Debeb et al. [82]
Liver metastasis										
miR-93	In vitro and in vivo	NR	Liver tissue, MDA-MB-231, T-47D, MCF7, and HEK293 cells	NR	qRT-PCR	NR	NR	NR	Metastasis suppressor	Shibuya et al. [83]

NR = Information not reported in the article.

## Data Availability

Not applicable.

## Supplementary Materials

The following supporting information can be downloaded at: https://www.mdpi.com/article/10.3390/cells12040525/s1. Table S1: Impact of microRNAs on cancer-derived metastatic phenotype [34,58,59,60,61,62,63,64,65,66,67,68,69,70,71,72,73,74,75,76,77,78,79,80,81,82,83,95,97,98,100].
