# Supplementary material for: Potential miRNA Use as a Biomarker: From Breast Cancer Diagnosis to Metastasis
Source: Cells. 2023 Feb 6;12(4):525. doi: 10.3390/cells12040525 (PMC9954167; doi:10.3390/cells12040525)
Supplement: Supplementary file 1 [file cells-12-00525-s001.zip › cells-2080802-supplementary.pdf]

cells MDPI

Table S1. Impact of microRNAs on cancer-derived metastatic phenotype [34,58–83,95,97,98,100].

miRNA	Study type	Samples number	Samples type	Samples sources	Detection methodology	AUC	Sensitivity	Specificity	Potential as	Reference
Let-7b-5p, miR-122-5p, miR-146b-5p, miR-210-3p and miR-215-5p	Multi-phase validation	289 BC samples and 257 normal controls	Blood samples	Hospital of Nanjing Medical University.	exiqon miRNA qPCR	0.843	81.1%	NR	Diagnosis	Li et al. [58]
miR-9 and miR-34a	Case-Control	31 tumour tissues, 31 adjacent non-tumour and 20 healthy controls	Tissues	National Tumor Bank and Isfahan Cancer Research Center of Seyed-o-Shohada Hospital.	qRT-PCR	0.71 and 0.72	83.33% and 72%.	70.37% and 76%	Diagnosis	Orange and Motovali-Bashi [59]
miR-373	Case-Control	196 newly diagnosed BC patients, 76 patients with benign breast lesions, and 49 healthy control	Blood samples	National research center.	RT-qPCR	0.98	90.8%	98.4%	Diagnostic and prognostic	Bakr et al. [60]

miR-185-5p and miR-362-5p	Case-Control	68 BC patients and 13 controls	Blood samples	Qilu Hospital of Shandong University.	RT-qPCR	0.957	92.65%	92.31%	Diagnosis	Zhang et al. [61]
miR-145	Meta-Analysis	8 reports (800 BC samples, 656 paracancerous and normal breast samples)	Tissues	NR	qRT-PCR	NR	NR	NR	Diagnosis	Lv. et al. [34]
miR-126-5p, miR-144-5p, miR-144-3p, miR-301a-3p, miR-126-3p, miR-101-3p, and miR-664b-5p	Clinical trial	21 patients with TNBC diagnosis and 21 healthy.	Blood samples	Medical Faculty of the Friedrich-Alexander University Erlangen-Nürnberg.	RT-qPCR	0.814	83.8%	74.2%	Diagnosis	Kahraman et al. [62]
miR-1246 and miR-21	*in vitro* and Case-Control	16 BC samples and 16 healthy samples	Exosomes from plasma	USA	Small RNA sequencing and qRT-PCR	0.73	NR	NR	Diagnosis	Hannafon et al. [63]
miR-1246 and miR-21	Meta-Analysis	11 reports	peripheral blood samples	NR	ELISA and qRT-PCR	NR	NR	NR	Diagnosis and prognosis	Wang et al. [64]

miR-21 and miR-27a	Case-Control	129 BC patients and 50 patients with benign breast lesions as control group	Fasting venous blood	Jinan People's Hospital Afliated to Shandong First Medical University.	fqPCR	0.737 and 0.771,	90.62%	74.00	Diagnosis	Li et al. [65]
Let-7b-5p, miR-106a-5p, miR-19a-3p, miR-19b-3p, miR-20a-5p, miR-223-3p, miR-25-3p, miR-425-5p, miR-451a, miR-92a-3p, miR-93-5p, and miR-16-5p	Four phase validation study	216 BC patients samples and 214 normal control samples	Blood samples	Jiangsu Provincial People Hospital.	exiqon miRNA qPCR validated by qRT-PCR	0.941	0.872	0.893	Diagnosis	Zou et al. [66]
miR-9-5p, miR-34b-3p, miR-1-3p, miR-146a-5p, miR-20a-5p, miR-34a-5p, miR-125b-5p	Cohort	135 Invasive Ductal Carcinoma patients and 125 healthy controls	Blood samples	Shenzhen Hospital, Peking University.	qRT-PCR	0.880	81.25%	86.25%	Diagnosis	Chen et al. [67]
Bone metastasis										
miR-21	*in vitro* and *in vivo*	8 mice per group	Bone tissues	NR	RNA-seq. qRT-PCR, western blot, confocal microscopy, and RNA interfering	Non reported (NR)	NR	NR	Promoting metastasis	Yuan et al. [95]

miR-19a	*in vitro* and *in vivo*	NR	BC tissue from bone-metastatic lesions and pri mary BC tissue	NR	Taqman PCR	NR	NR	NR	Promoting metastasis	Wu et al. [68]
miR-20a-5p	*in vitro*	NR	MCF-7 and MDA-MB-231 cell lines	NR	qRT-PCR	NR	NR	NR	Promoting migration and invasion	Guo et al. [69]
miR-16, miR-133a and miR-223	*in vitro* and *in vivo*	NR	MDA-MB-231 cell line, bone and tibia samples	NR	RT-qPCR	NR	NR	NR	Promoted osteoclast activities and bone destruction	Kitayama et al. [70]
miR-143	*in vitro*	15 patients	BC tissue, normal adjacent tissues, MDA-MB-231, MDA-MB-436, SK-BR3, CAMA-1 and normal MB 157 cell lines	Fudan University Shanghai Cancer Center.	qRT-PCR	NR	NR	NR	Tumour suppressor	Du et al. [71]
miR-30b-5p	cohorts	20 localized tumours and 25 advanced disease	Liquid biopsies	Breast Cancer Clinic and Laboratory Medicine Department of the Portuguese Oncology Institute of Porto.	qRT-PCR	0.831	88.9%	66.7%	Progression biomarker	Estevão-Pereira et al. [72]

Lung metastasis										
miR-663, miR-210, miR-1, miR-301a, miR-135b, miR-451, miR-30a and miR-199a-5p	*in silico*	439 samples	Clinical data	NR	METABRIC and TCGA databases	0.774	NR	NR	Metastasis to lung	Zhang at al. [97]
Let-7s family	Cohort, *in vitro* and *in vivo*	Cohort I) 30 cases<br>Cohort II) 140 cases<br>Cohort III) 145 cases	Tumour tissues	Shanghai Institute of Nutrition and Health Sciences.	qRT-PCR	NR	NR	NR	Poor prognosis and lung metastasis	Qi et al. [98]
miR-138-5p	*in vitro* and *in vivo*	NR	Lung tissues	Chinese PLA General Hospital.	RT-qPCR	NR	NR	NR	Promotion of lung metastasis and progression	Xun et al. [73]
miR-934	case-control and *in vitro*	50 pairs of frozen BC tissue, adjacent normal tissue and 21 lymph node metastasis BC tissue samples	Lung metastatic tissue	Huai'an Maternity and Child Health Care Hospital.	qPCR	NR	NR	NR	Prognosis biomarker	Lu et al. [74]

miR-4731	*in vitro* and *in vivo*	50 patients	Tumour tissue, MDA-MB-436, MDA-MB-453, MCF-7, and MDA-MB-231 cell lines	Chongqing University Central Hospital.	RT-qPCR	NR	NR	NR	Inhibition of EMT transition	Lang et al. [75]
miR-18a	*in vitro* and *in vivo*	NR	Primary tumour and lung tissues	NR	qPCR	NR	NR	NR	Promotion of metastasis	Krutilina et al. [76]
Brain metastasis										
miR-4428 and miR-4480	Case-Control	51 samples brain metastasis and 28 samples without brain metastasis	Serum	National Cancer Center Hospital. Japan.	Microarray	0.779 and 0.781 respectively	82.4% and 76.5%	f 64.3% and 71.4%	Prediction of brain metastasis	Sato et al. [77]
miR-10b	Retrospectiv e Case-Control	20 samples with brain metastasis and 10 samples without brain metastasis	Tumour tissues block	Department of Pathology, Karmanos Cancer Institute, Wayne State University.	qRT-PCR	NR	NR	NR	Biomarker to brain metastasis and a potential therapeutic target	Ahmad et al. [78]
miR-211	*in vitro* and *in vivo*	NR	Brain tissues	NR	qRT-PCR	0.932	NR	NR	Brain metastasis predictor	Pan et al. [100]

miR-26b-5p and miR-101-3p	Pre-Clinical	NR	Brain metastatic tumours and primary tumours	NR	RT-qPCR	NR	NR	NR	Prognostic and therapeutic	Harati et al. [79]
miR-125a	Cohort	Cohort I) 13 pairs of BC and adjacent normal breast tissues Cohort I1) 6 BC brain metastasis (BCBM) tissues Cohort III) 20 BC and 20 BCBM patients' plasma samples, and Cohort IV) 53 BCBM patients' primary tumour tissues.	Tissues and plasma	Liaocheng People's Hospital.	RT-qPCR	NR	NR	NR	Therapeutic target	Fu et al. [80]
MN-anti-miR10b	*in vitro* and *in vivo*	NR	NR	NR	Bioluminescence and Microscopy	NR	NR	NR	Treatment in brain metastasis	Yoo et al. [81]
miR-141	*in vitro* and *in vivo*	10-15 mice per group	Brain metastases tissues	NR	RT-PCR and fluorescence methods	NR	NR	NR	Biomarker and therapeutic target of brain metastasis	Debeb et al. [82]
Liver metastasis										

miR-93	*in vitro* and *in vivo*	NR	Liver tissue, MDA-MB-231, T-47D, MCF7 and HEK293 cells	NR	RT-qPCR	NR	NR	NR	Metastasis suppressor	Shibuya et al. [83]

NR= Information non reported in the article.
